# Distinct infectivity and neutralization antibody responses in the highly homologous AAV Go.1 and AAV5

**DOI:** 10.3389/fmed.2025.1554449

**Published:** 2025-04-04

**Authors:** Mei Li, Haixiao Ma, Yang Wu, Yunling Gao, Jie Wang, Hanbing Wang

**Affiliations:** ^1^Department of Anesthesiology, First People Hospital of Foshan, Foshan, China; ^2^Institute of Neuroscience and Brain Diseases; Xiangyang Central Hospital, Affiliated Hospital of Hubei University of Arts and Science, Xiangyang, China; ^3^State Key Laboratory of Magnetic Resonance and Atomic and Molecular Physics, National Center for Magnetic Resonance in Wuhan, Wuhan Institute of Physics and Mathematics, Innovation Academy for Precision Measurement Science and Technology, Chinese Academy of Sciences-Wuhan National Laboratory for Optoelectronics, Wuhan, China; ^4^Department of Radiology, Songjiang Research Institute, Shanghai Key Laboratory of Emotions and Affective Disorders, Songjiang Hospital Affiliated to Shanghai Jiao Tong University School of Medicine, Shanghai, China

**Keywords:** gene therapy, rAAV Go.1, cell transfection, neutralizing antibody, packaging efficiency

## Abstract

**Introduction:**

Goat-derived adeno-associated virus (AAV) vectors, such as AAV Go.1, represent a novel platform for gene therapy due to their unique origin and potential advantages in transduction efficiency and immune evasion. However, their therapeutic potential and biological properties remain underexplored.

**Methods:**

In this study, we developed a recombinant AAV (rAAV) Go.1 by replacing the goat AAV rep gene with the standard AAV2-rep gene to improve packaging efficiency. We compared the transduction efficiency of rAAV Go.1 with that of AAV5, a closely related serotype with 95% genome similarity, both *in vitro* and *in vivo*. Additionally, we assessed immune evasion properties by evaluating resistance to neutralization using sera from rAAV5-immunized mice and human volunteers. To further enhance transduction efficiency, we introduced site-specific mutations in the VP1 unique (VP1u) region and VP1/2 common region.

**Results:**

The rep gene modification led to a significantly higher packaging efficiency for rAAV Go.1 compared to the original goat AAV. rAAV Go.1 exhibited markedly higher transduction efficiency than AAV5 in both in vitro and in vivo models. Furthermore, rAAV Go.1 demonstrated a 4-fold increase in resistance to neutralization by sera from rAAV5-immunized mice. A study involving 20 healthy volunteers revealed that high-titer neutralizing antibodies had a more pronounced inhibitory effect on rAAV5 compared to rAAV Go.1. Mutagenesis studies identified key modifications that enhanced viral properties: K32R, K91R, and K122R mutations in the VP1u region significantly improved viral production, while K137R (VP1u) enhanced transduction efficiency *in vitro* and *in vivo*.

**Discussion:**

Our findings highlight the potential of rAAV Go.1 as an improved gene therapy vector with superior transduction efficiency and enhanced immune evasion. The identified VP1 mutations further optimize viral properties, making rAAV Go.1 a promising candidate for future therapeutic applications.

## 1 Introduction

Adeno-associated viral vectors (AAVs) have emerged as the promising candidates for gene therapy. Adeno-associated virus (AAV)-based gene therapy has emerged as a highly effective and.

versatile tool for treating a wide range of genetic disorders (%^1^–^3^). One of its key advantages is its excellent safety profile, as AAV is naturally non-pathogenic and replication-deficient, significantly reducing the risk of adverse effects in patients (4). Furthermore, AAV can mediate long-term gene expression (5), particularly in non-dividing cells such as neurons and cardiac tissues (6), due to its ability to persist episomally without integrating into the host genome (7), thereby minimizing the risk of insertional mutagenesis (8). AAV also offers broad tissue-targeting capabilities through its diverse serotypes, each exhibiting unique tropisms. Major challenges with current rAAV vectors include pre-existing human antibodies, inability to selectively target specific cells or tissues, lack of standardization in vector titers and potency, and limited packaging capacity (~5 kb for ssAAV and ~2.5 kb for scAAV) (4, 9). Exploring new AAV serological strategies has become an important direction in gene therapy research and development.

Naturally occurring wild-type Adeno-associated viruses (wtAAVs) are rapidly evolving, with over 1,000 variants identified from diverse sources, including adenovirus stocks, human and non-human primate tissues, as well as other mammalian and non-mammalian species (%^10^–^15^). Birds are examples of non-mammalian sources that contribute to the vast genomic diversity of AAVs (16). These species provide unique AAV variants with distinct genetic sequences and capsid structures (17), which offer valuable insights into the evolutionary trajectory of AAVs and expand the pool of vectors available for gene therapy applications. Such non-mammalian-derived AAVs may possess novel characteristics, including altered tissue tropism and reduced cross-reactivity with pre-existing antibodies in humans, making them promising candidates for next-generation gene delivery systems.

The AAV Go.1 genome, isolated from a goat, shares 95% sequence identity with the human AAV5 genome, while the capsid proteins exhibit a high homology of 94%. Comparative analysis of the Rep open reading frame reveals a 99% amino acid identity between AAV5 and AAV Go.1. Additionally, the inverted terminal repeats (ITRs) of AAV Go.1 share 100% sequence identity with those of AAV5. Both the assembly-activating protein (AAP) and the membrane-associated AAV protein (MAAP) exhibit 100% amino acid identity with AAV5 (%^18^–^21^). Notably, most of the 42 amino acid differences are localized on the exterior surface of the capsid, which plays a critical role in host interactions. Some of these variations overlap with the binding sites of known AAV5 antibodies, and studies have demonstrated that AAV5 antibodies exhibit stronger binding affinity to AAV5 than to AAV Go.1 (22). Furthermore, previous investigations utilizing human intravenous immunoglobulin (IVIG) have confirmed that AAV Go.1 possesses lower immunogenicity compared to AAV5. However, further experiments using human sera are necessary to comprehensively assess the neutralizing antibody titers specific to AAV Go.1.

Adeno-associated viral (AAVs) vectors have demonstrated efficacy in clinical trials for treating conditions such as Leber congenital amaurosis and hemophilia B (%^23^–^25^). The coding regions of AAV comprise two primary open reading frames (ORFs), known as *rep* and *cap*, which are essential for viral replication and DNA packaging. The Cap ORF encodes phenotypes relevant to tissue tropism and immune recognition. The AAV capsid structure consists of 60 subunits of viral proteins VP1, VP2, and VP3. Notably, VP1 is required for viral infectivity, indicating the presence of functional domains within the unique VP1 region (26, 27). This region contains a phospholipase A2 (PLA) domain and nuclear localization signals (NLS), which are necessary for infection at a post-entry step, including endosome release (%^28^–^30^).

Here, we aim to evaluate the effectiveness of AAV Go.1 and AAV5 as vectors for gene therapy, with a focus on transduction efficiency and immunogenicity. We observed that the packaging efficiency of AAV Go.1 is higher when using the replicase (*rep*) gene from AAV 2 instead of its native replicase gene, AAV Go.1 *rep*. The delivery of AAV Go.1 was evaluated for both transduction efficiency and the host immune response, compared with AAV5 both *in vitro* and *in vivo*. AAV Go.1 showed a significant improvement in cell transfection for gene expression compared to AAV5 *in vitro* and *in vivo*. In an *in vitro* neutralization assay using mouse serum, AAV Go.1 exhibited higher resistance than AAV5 (100-fold). In our study involving 20 healthy volunteers, we noted that higher titers of neutralizing antibodies corresponded to a greater inhibitory impact on the cellular transduction efficiency of AAV5. It indicates that AAV Go.1 may have advantages over AAV5 in terms of evading neutralizing antibodies present in the host immune system. Furthermore, the VP1 and VP2 N termini played an important role in AAV infection. K32R, K91R and K122R mutations of VP1 and VP2 N termini enhanced virus production, while K137R and K142R improved cell transduction. Overall, based on considerations of tissue tropism, immunogenicity, and safety, AAV Go.1 appears to be the more suitable AAV vector for specific gene therapy applications when compared to AAV5.

## 2 Materials and methods

### 2.1 Animal ethics statement

Wild-type male C57BL/6 mice (6–8 weeks old, *n* = 3/group) were purchased from Hunan SJA Laboratory Animal Co., Ltd. (Changsha, Hunan, China). All animals were kept in a temperature-controlled environment with a constant 12-h light–dark cycle and randomization was applied to allocate animals to experimental groups. Food and water were provided *ad libitum*. The care and use of all animals were conducted in accordance with the guidelines established by the Guang Dong Medical Laboratory Animal Center, which granted ethical approval (C202309-67). Clinical samples consisted of normal human serum samples were collected from 20 healthy people at the physical examination center of Xiangyang Central Hospital (NO: 2023-117).

### 2.2 Cell and culture condition

The HEK293T and HeLa cells were cultured in Dulbecco’s modified Eagle’s medium supplemented with 10% fetal bovine serum and 1% penicillin–streptomycin. All cell lines were maintained at 37°C with 5% CO_2_.

### 2.3 Construction of virus packaging plasmids

The AAV5 capsid gene of pAAV-RC2/5 (composed of AAV 2 *rep* and AAV5 *cap* genes) were removed by digestion with AflII and SbfI (New England Bioland, MA). The AAV Go.1 caspid coding region was created with PCR, and the PCR fragment was fused into the digested pAAV-RC2/5 plasmid to generate a new plasmid of pAAV-RC2/Go.1. The *rep* gene of AAV Go.1 was inserted into the pAAV-RC2/Go.1 by replacing the AAV2 *rep* gene to generate pAAV-RC Go.1 plasmid. Site-directed mutagenesis of the N-terminal of VP1 in the AAV Go.1 capsids encoded gene was performed using a two-stage PCR (31). Primers were designed to introduce changes from Lysine (AAG) to Arginine (CGG) for each of the mutated residues. Furthermore, this study generated AAV plasmids pAAV-CMV-Luciferase-2A-EGFP-WPRE-poly. All constructs were confirmed by sequencing.

### 2.4 Production of recombinant AAV vectors and virus titration

The rAAV virions were produced in HEK293 cells using the traditional triple-plasmid transfection method (32). Briefly, HEK293 cells at 80% confluence were cotransfected with pAAV-RC, pAAV-luciferase-2A-EGFP, and pAAV-helper using PEI reagent. Cells were harvested after 72 h post-infection and the viruses were purified through an iodixanol step using the density gradient centrifugation, as described previously (32). Titers of the purified AAV virions were determined by qPCR with SYBR Green PCR Master Mix (Bio-Rad) using the following primer pairs specific for the WPRE: F5′-TCCCATAGTAACGCCAATAGG-3′, R5′-CTTGGCATATGATACACTTGATG-3′. Standard curves were established with 10-fold serial dilutions of standard plasmids. The titers of AAV pseudo-vectors and the capsid composition of AAV vectors were also assessed through silver staining analysis. In brief, AAV vectors (at a concentration of approximately 1 × 10^10^ particles) were separated by electrophoresis on a 10% SDS-PAGE gel, and the standard silver staining procedures outlined by the manufacturer (Pierce Silver Stain Kit, Thermo Scientific) were followed.

### 2.5 Western blotting

Viruses were lysed in radioimmunoprecipitation assay (RIPA) buffer and the samples were separated by sodium dodecylsulfate-polyacrylamide gel electrophoresis (SDS-PAGE), followed by transferring onto polyvinylidene fluoride (PVDF) membranes. The membranes were probed with an anti-VP mouse monoclonal B antibody (1:1,000; 65,158-lg, proteintech) for 24 h at 4°C and subsequently washed three times with 0.1% Tween 20/PBS, followed by incubation for 1 h with horseradish peroxidase (HRP)-conjugated goat anti-mouse secondary antibody (1:10,000; ab205719, Abcam). The membranes were washed three times with 0.1% Tween 20/PBS and visualized by exposure to FluorChem HD2 Imaging System (Alpha Innotech) after the addition of a chemiluminescent substrate (SuperSignal West Dura Extended Duration Substrate; 34,075; Thermo Scientific Pierce).

### 2.6 Luciferase assay

Briefly, 1 day prior to infection, cells were seeded at 10^4^ per well in a 96-well plate with MEM containing 10% FBS, penicillin, and streptomycin (both at 100 U/ml) and incubated at 37°C with 5% CO_2_ for 48 h. This experiment was performed in triplicates. Then, the AAVs were added to the pre-seeded cells. After 48 h, the medium was removed and the cells were washed and lysed. Luciferase activity was measured using a luciferase assay kit, according the manufacturer’s instructions (Promega, Madison, USA). All determinations were completed in duplicate, and the background luciferase activity was subtracted from uninfected cells.

### 2.7 Bioluminescence imaging

The two 8-week-old mice were injected with AAV5 or AAV Go.1 (10^11^ vg/mouse) through the tail vein, respectively. After 3 weeks, the infected mice underwent bioluminescence imaging. Firstly, the mice were anesthetized and maintained on 1–1.2% isoflurane in oxygen. The D-Luciferin substrate (Promega, Madison, USA) was diluted at a 1:20 ratio in PBS, and each mouse was intraperitoneally (*i.p.*) injected with 100 μl of this mixture. Ten minutes following D-luciferin administration, all mice were imaged using an IVIS CCD camera system (Caliper Life Sciences, Hopkinton, USA) and subsequently processed with Living Image software (version 4.5, Caliper Life Sciences). Subsequently, the mice were euthanized and the kidneys were explanted and scanned with the IVIS (Caliper Life Sciences).

### 2.8 Mouse immunization and serum antibody preparation

Male C57BL/6 mice (6–8 weeks old, *n* = 3) were initially anesthetized with isoflurane (3.0–4.0%), and 100 μl rAAV2/5 -CMV-Luciferase-2A-EGFP-WPRE-polyA was infused to their bodies through their lateral tail vein at a dose of 5 × 10^11^ vg/mouse. After 4 weeks, blood was collected *via* the inferior vena cava. Serum was obtained after allowing whole blood to coagulate at room temperature for at least 30 min, followed by centrifugation at 1,000 *g* for 10 min. At the termination of each experiment, mice organs were snap-frozen and stored at −80° C.

### 2.9 Cell-based *in vitro* AAV transduction inhibition assay

An *in vitro* AAV transduction inhibition assay was used to quantify the amount of anti-AAV neutralizing serum in mouse and human serum. The serum supernatant was stored at −80°C and heat-inactivated at 56°C for 30 min before the assay. HEK293T cells were seeded in poly-L-lysine coated white 96-well plates (Corning) at 20, 000 cells/well 24 h before the addition of the virus. DMEM-alpha media containing 1% Pen-strep was used to dilute mouse serum from 1:10 to 1:640 by 2-fold serial dilutions in duplicate. AAV5 or AAV Go.1 virus-containing metridia luciferase transgene at the same multiplicity of infection (MOI) was incubated with the various serum dilutions at 37°C for 1 h, then added to cells. 48 h after virus addition, media was aspirated, and cells were lysed and assayed for metridia luciferase expression as described above. Positive controls with only virus and no serum and negative controls with no virus and no serum were included in the plate read. The neutralizing antibody titer (NAb titer) is defined as the reciprocal of the dilution at which 50% of rAAVs transduction is inhibited. To quantify the anti-AAV antibody, the transduction vs. serum dilution curves were fit using four parameters logistic regression in Prism 9 (GraphPad).

### 2.10 Statistical analysis

We compared mean values from different experimental groups using a two-tailed Student’s t-test or one-way analysis of variance. For all statistical analyses, an unpaired t-test was used to compare the chemiluminescence signals from corresponding AAV pre-incubated with tested serum with the control of AAV in the presence of the diluent only. Data were considered significant when *p* values were <0.05.

## 3 Results

### 3.1 Vector design and production

**Figure 1:**
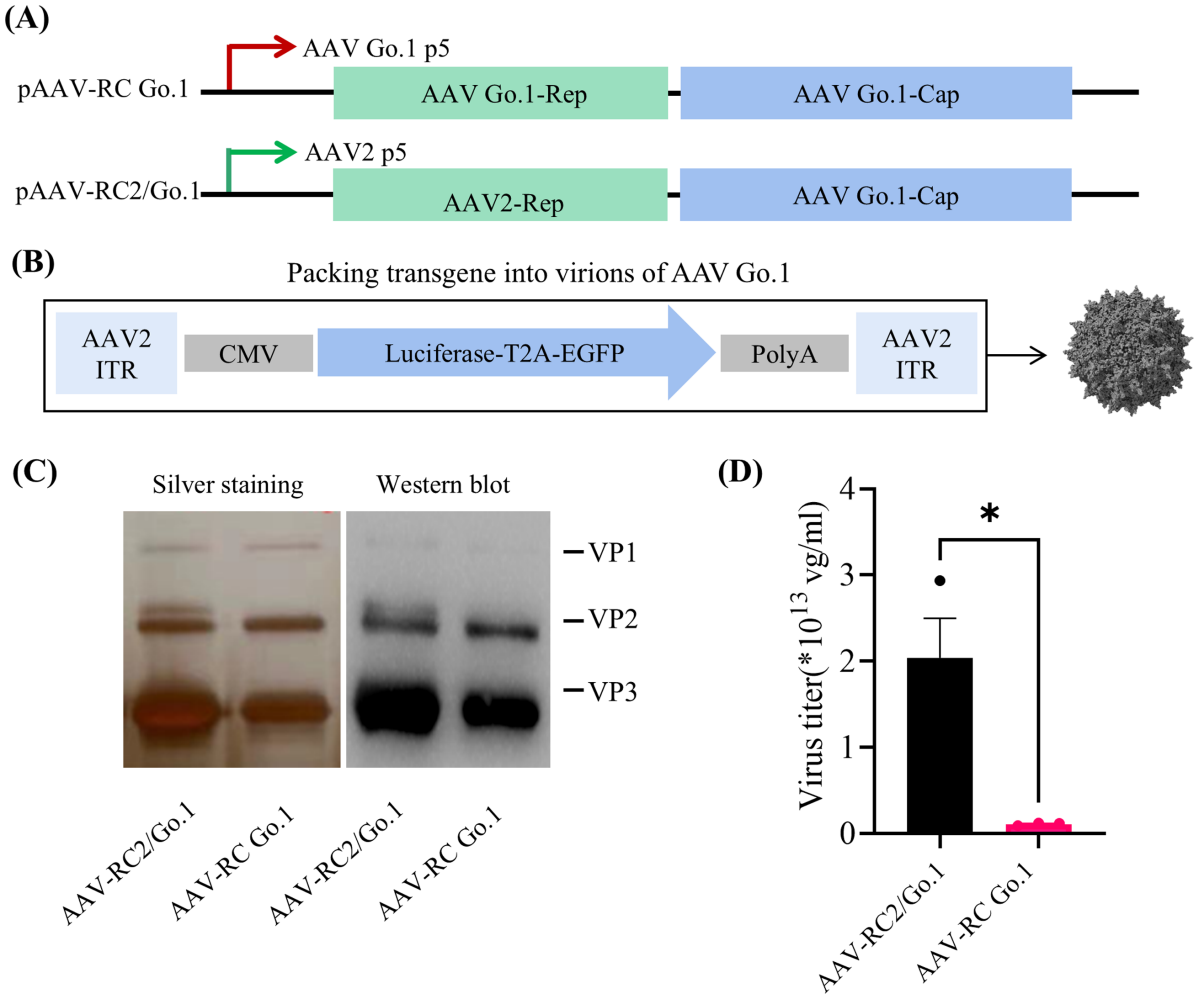
Study design and virus production. **(A)** Overview the utilized the *rep/cap* helper plasmid, pAAV2/Go1 contain AAV2 P5 promoter (green arrow) and *rep* element. The pAAV Go/Go1 derived from AAV Go1 P5 promoter (red arrow) and *rep* gene; **(B)** Diagram of packing vector containing AAV2-ITRs, the cytomegalovirus (CMV)-driven luciferase and EGFP transgenes; **(C)** SDS-PAGE gels with silver staining and Western blotting. Anti-AAV VP1/VP2/VP3 (clone B1) antibody was used to visualized different viral proteins (VP) in Western blotting; **(D)** Comparison of genome titers of two rAAVs which were determined by qPCR. The data were shown using the mean + SEM for three independent experiments.

The AAV Go.1 capsid gene was pseudotyped by cloning it into rAAV-RC plasmids containing either the AAV2 or Go.1 rep gene under the control of the P5 promoter (Figure 1A). The packaging vector included a luciferase gene driven by the cytomegalovirus (CMV) immediate-early promoter/enhancer, flanked by AAV2 inverted terminal repeat (ITR) sequences (Figure 1B). Previous studies employed a similar approach by pseudotyping the AAV Go.1 capsid gene with rAAV-RC plasmids containing the AAV2 *rep* gene and the P5 promoter (22). To compare the performance of these two packaging plasmids in terms of the *rep* gene and P5 promoter, silver staining was performed to assess total capsid protein expression (VP1, VP2, and VP3). The results indicated that rAAV Go.1 constructs with the Go.1 *rep* gene exhibited lower capsid protein expression compared to constructs containing the AAV2 *rep* gene (Figure 1C). Western blot analysis, using specific antibodies, confirmed the identity of VP1, VP2, and VP3, showing similar expression patterns between the two constructs, with VP3 displaying the strongest band relative to VP1 and VP2. Vector genome (vg) titers further revealed that constructs using the pAAV-RC2/Go.1 plasmid produced significantly higher titers than those with the pAAV-RC Go.1 plasmid (Figure 1D). These results collectively demonstrate that pairing the AAV Go.1 capsid with the AAV2 Rep element improves capsid production and vector yield, making it the optimal configuration for subsequent experiments. In the following experiments, all rAAV-RC2/Go.1 used are represented as rAAV Go.1, ensuring consistency in the description and highlighting the focus on this specific recombinant vector throughout the study.

### 3.2 Comparison of rAAV5 and rAAV Go.1 mediated transgene expression

**Figure 2:**
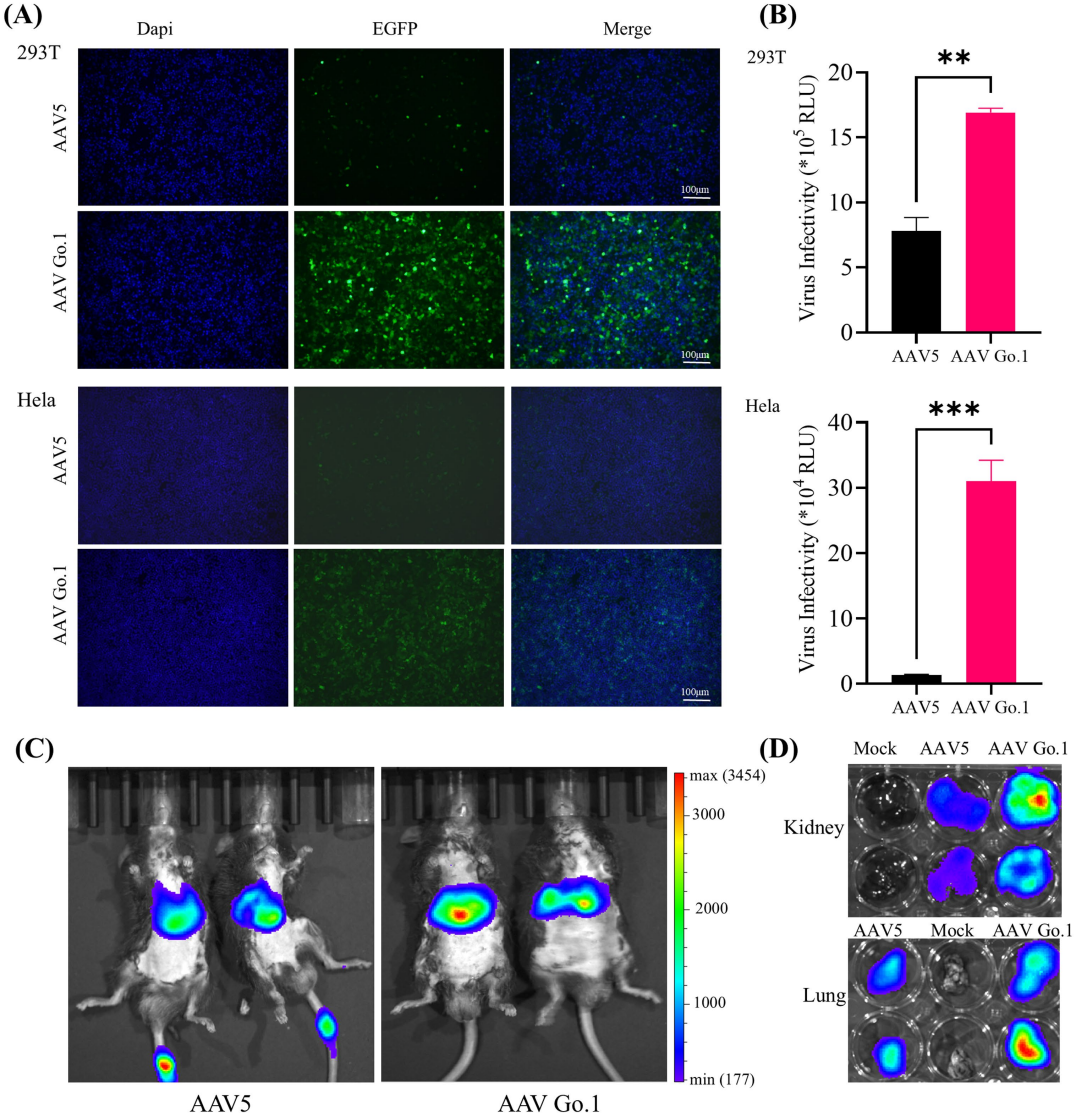
Comparison of the transduction in cells and animals between rAAV Go.1 and rAAV5. **(A,B)** HEK293T or HeLa cells were incubated with rAAV5 or rAAV Go.1 at a MOI of 10^5^, respectively. At 48 h post-infection, the green cells with were visualized by an Olympus microscope, and the representative confocal images from three independent experiments are illustrated (Scale bar: 100 μm). **(B)** The luciferase activities are presented as mean + SD for three independent experiments. **(C)** *In vivo* imaging of luciferase activity was performed 3 weeks after administration of rAAV5 and rAAV Go.1. Representative ventral views of the results are shown. **(D)** Representative image of the explanted kidneys and lungs scanned with IVIS.

Previous studies indicated that there were no significant differences in transduction efficiency between the two recombinant vectors in 293 and Hela cells. However, rAAV Go.1 demonstrated a greater transduction efficiency *in vivo* compared to rAAV5. To further validate the transduction efficiency of rAAV Go.1, comparative experiments were conducted under identical conditions against rAAV in both cellular models and *in vivo* animal models. Cell transduction efficiency was initially assessed in HEK293T and HeLa cells, which were infected with 10^5^ vector genomes (vg)/cell. At 48 h post-infection, GFP expression was visualized and quantified using fluorescence microscopy. The results showed that rAAV Go.1 significantly increased the number of GFP-positive cells compared to rAAV5 in both cell lines (Figure 2A). To further confirm transduction efficiency, luciferase activity was measured at 48 h post-infection, and rAAV Go.1 demonstrated a significant enhancement in luciferase expression compared to rAAV5 (Figure 2B). *In vivo* transduction efficiency was evaluated by injecting 10^11^ vg of each vector into mice via the tail vein. Three weeks post-injection, rAAV Go.1 targeted the liver with tropism similar to rAAV5 but achieved significantly higher transgene expression levels (Figure 2C). Additionally, luciferase expression was observed in the kidney and lung for both rAAV5 and rAAV Go.1, with rAAV Go.1 displaying superior expression up to 3 weeks post-injection (Figure 2D). These findings collectively demonstrate that rAAV Go.1 significantly enhances gene expression in both *in vitro* and *in vivo* models, suggesting its potential as an improved vector for gene therapy compared to the conventional rAAV5.

### 3.3 Evaluating neutralization of rAAV5 and rAAV Go.1 by anti-AAV5 antibodies

**Figure 3:**
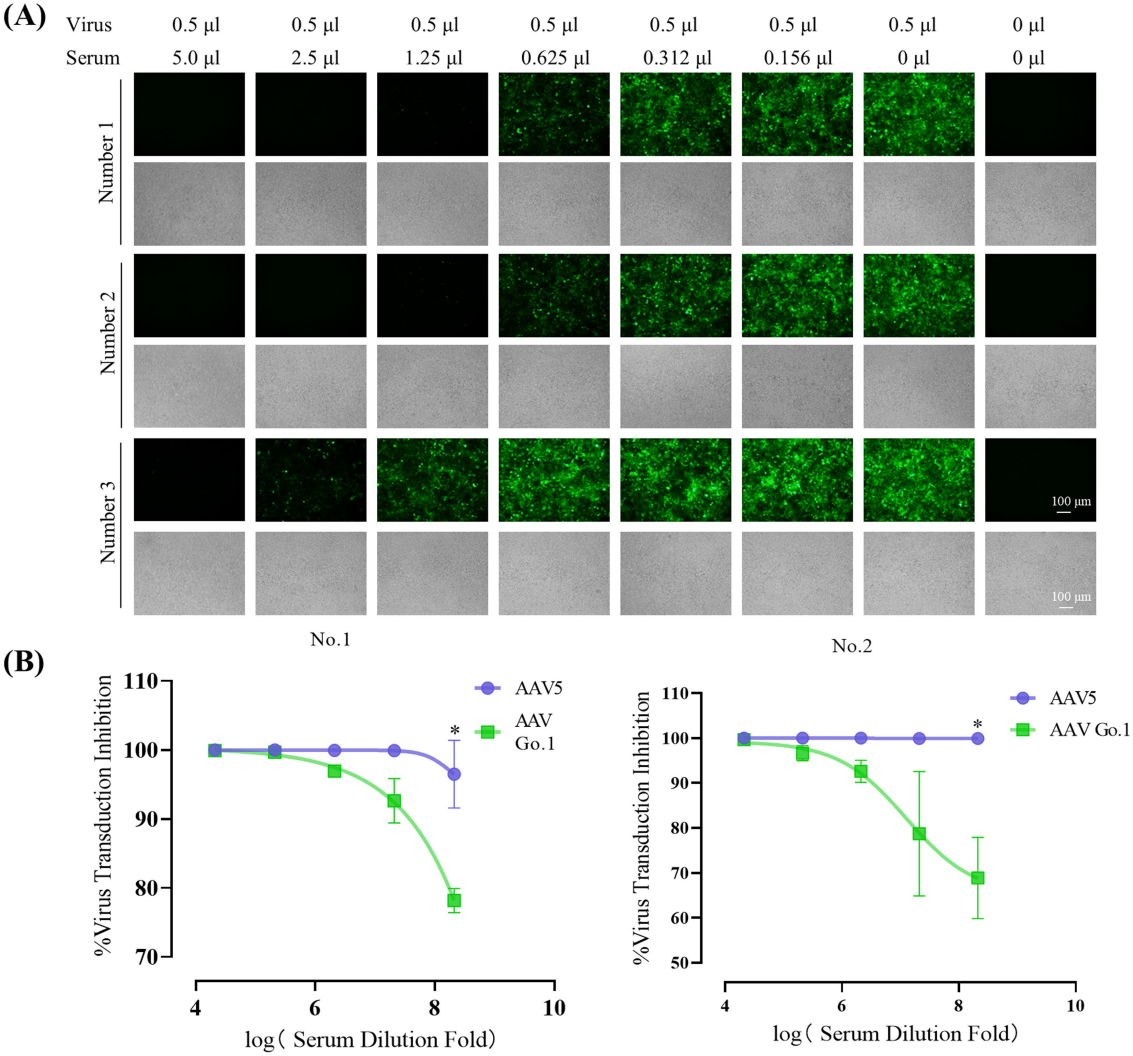
Inhibitory effect of anti-AAV5 antibodies on AAV Go.1 Transduction. **(A)** Serum from the treated mice was collected 4 weeks after an injection of 1 × 10^11^ vg rAAV5 through lateral tail-vein. The rAAV5/luc vectors in 0.5 μl were incubated with equal volumes of different concentration of mouse serum or PBS. The mixture was then added into HEK293T cells. After 48 h of infection, the green fluorescent cells were visualized by an Olympus microscope (Scale bars: 100 μm). **(B)** Results of the *in vitro* NAb assay showed the inhibition of AAV5 and AAV Go.1 transduction in HEK293T cells by mouse serum. The AAV/luc vectors (20 μl) were incubated with equal volumes of different concentration of mouse or PBS. The mixture was then used to infect HEK293T cells in a 96-well plate. Luciferase activity was measured 48 h later. The inhibition was calculated as 100% minus the ratio of luciferase activity from the serum group to that of the PBS group. The dates are shown using mean + SEM for two independent experiments.

To evaluate the impact of anti-AAV antibody levels following rAAV5 vector administration, animals were infused with the rAAV5 vector expressing EGFP via the tail vein. Four weeks after transduction, blood samples were collected through the inferior vena cava under anesthetized. Serum was then collected and utilized to detect the anti-AAV antibody levels. Serial dilutions of each serum (1:10 to 1:320) from three different mice were utilized to detect the anti-AAV antibody levels of rAAV5. The expression of green fluorescence protein (GFP) following transduction with rAAV5-CMV-Luciferase-2A-EGFP at an MOI of 10^6^ was measured to evaluate neutralization (Figure 3A). For mice 1 and mice 2, GFP expression was nearly completely inhibited at a serum dilution of 1:80, indicating the generation of anti-AAV5 antibody through tail vein infusion of rAAV5. In contrast, in mouse 3, GFP expression inhibition occurred at a dilution of 1:40, suggesting lower ani-AAV5 antibodies levels compared to the other two animals. To assess the cross-reactivity of neutralizing antibodies against rAAV5 with AAV Go.1, serum samples from the first two animals were utilized.

In order to examine the effect of anti-AAV5 antibody on the transduction by AAV Go.1 vector *in vitro*, we preincubated luciferase vector of AAV Go.1 or AAV 5 at MOI of 10^4^ prior to transducing HEK293T cells with decreasing serum concentrations (1:10 to 1:160) from the first two animals (Figure 3B). The results showed that AAV5 antibodies in the serum could completely inhibit virus transduction of rAAV 5, even with a 320 times dilution. However, anti-AAV5 antibodies showed a low degree of cross reactivity with rAAV Go.1 (Figure 3B). Thus, these results indicate partial immune escape or reduced recognition by neutralizing antibodies.

### 3.4 Assessment of NAb against AAV Go.1 in human serum samples

**Figure 4:**
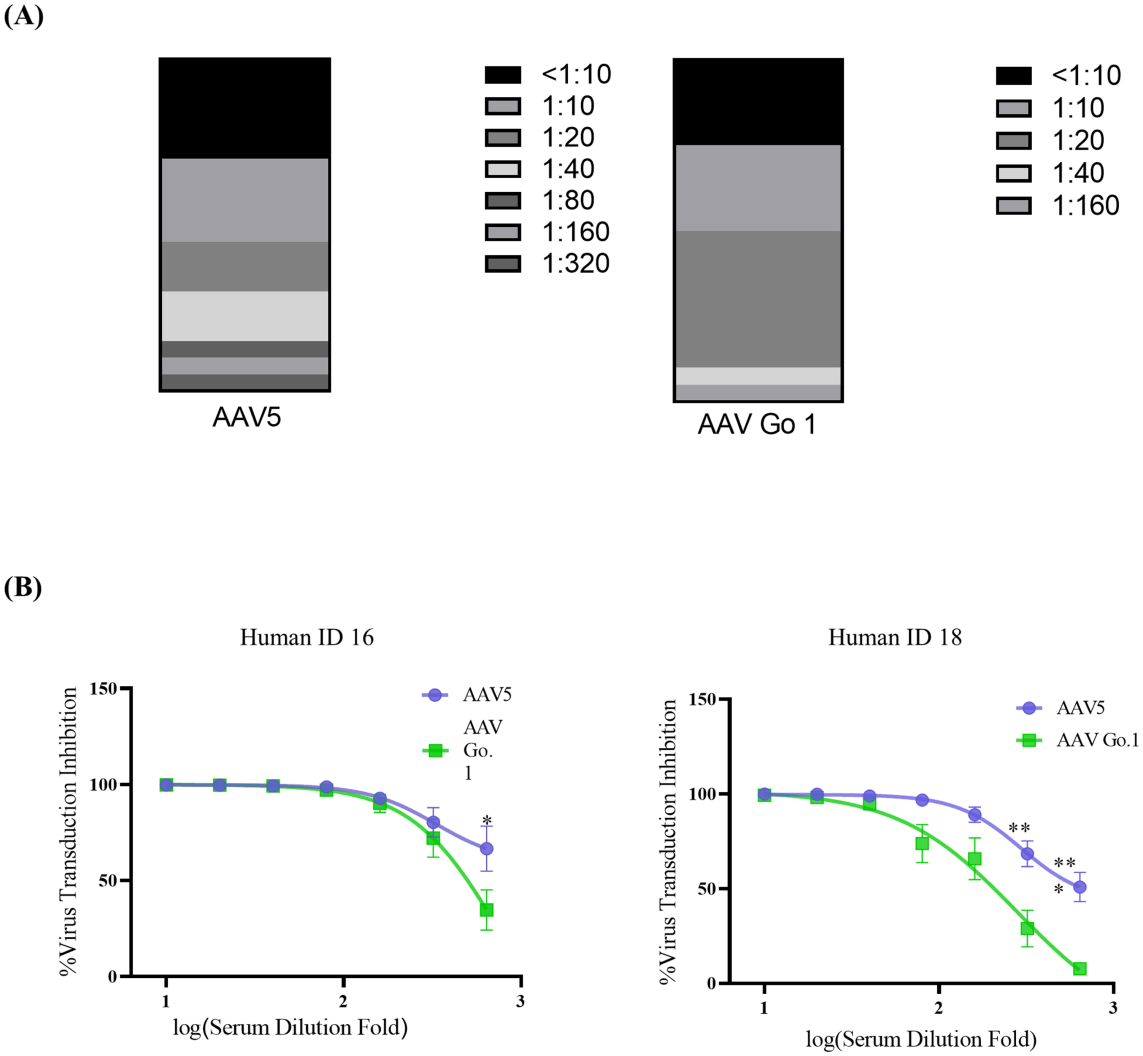
*In vitro* neutralizing antibody assay for AAV Go.1 and AAV5 using human serum **(A)** Neutralizing antibodies (NAbs) were determined using an *in vitro* transduction inhibition assay. The stacked histogram shows the distribution of serum samples with varying levels of AAV neutralizing activity. **(B)** The NAb testing for AAV5 and AAV Go.1 was performed with two human serum samples (No 16 and No 18). The dates were shown using mean + SEM for three independent experiments.

To further validate the immunogenicity of rAAV Go.1 in human, the NAb values of AAV Go.1 and rAAV5 were investigated with the serum samples from 20 healthy individuals. The evaluation procedures were similar to the mouse serum study. The NAb values for all the serum sample for rAAV5 and rAAV Go.1 were collected in Table 1 and Figure 4A. When the seropostive criteria were set at 1:40 and 1:640 dilutions, NAb against serotype rAAV5 were observed most frequently (Table 1). Samples 16 and 18 had NAb titer >640 against AAV5. The neutralizing antibody titer was much higher for rAAV5 compared to AAV Go.1. Among these human serum samples, two typical serum samples (16 and 18) showed much higher NAb for rAAV5 and AAV Go.1 (Table 1). They showed NAb values of rAAV Go.1 with 1:320 and 1:160, respectively, which were much lower than AAV5 (>1:640 and 1:320, respectively). To illustrate the detection procedures of the NAb for both viruses, a set of representative *in vitro*-transducing assays for serum samples 16 and 18 were illustrated in Figure 4B. These results demonstrated that AAV Go.1 was more efficient at escaping from human immunity. Based on the results of human and animal studies, mutations of rAAV Go.1 were implemented in the following study to screen for better virus vectors for gene therapy.

**Table 1:** The NAB test with different human serum samples for AAV5 and AAV Go.1.

Human ID	Anti-AAV5	Anti-AAV Go1	Human ID	Anti-AAV5	Anti-AAV Go1
1	1:10	1:10	11	<1:10	1:20
2	<1:10	<1:10	12	1:10	1:10
3	<1:10	<1:10	13	1:40	1:20
4	1:40	1:20	14	1:10	1:10
5	<1:10	<1:10	15	1:20	1:20
6	1:80	1:20	16	>1:640	1:320
7	1:20	1:20	17	<1:10	<1:10
8	1:20	1:20	18	>1:640	1:160
9	<1:10	<1:10	19	1:10	1:10
10	1:40	1:20	20	1:20	1:20

### 3.5 Multiple single point mutants in rAAV Go.1 VP1 protein mediate viral packing efficiency

**Figure 5:**
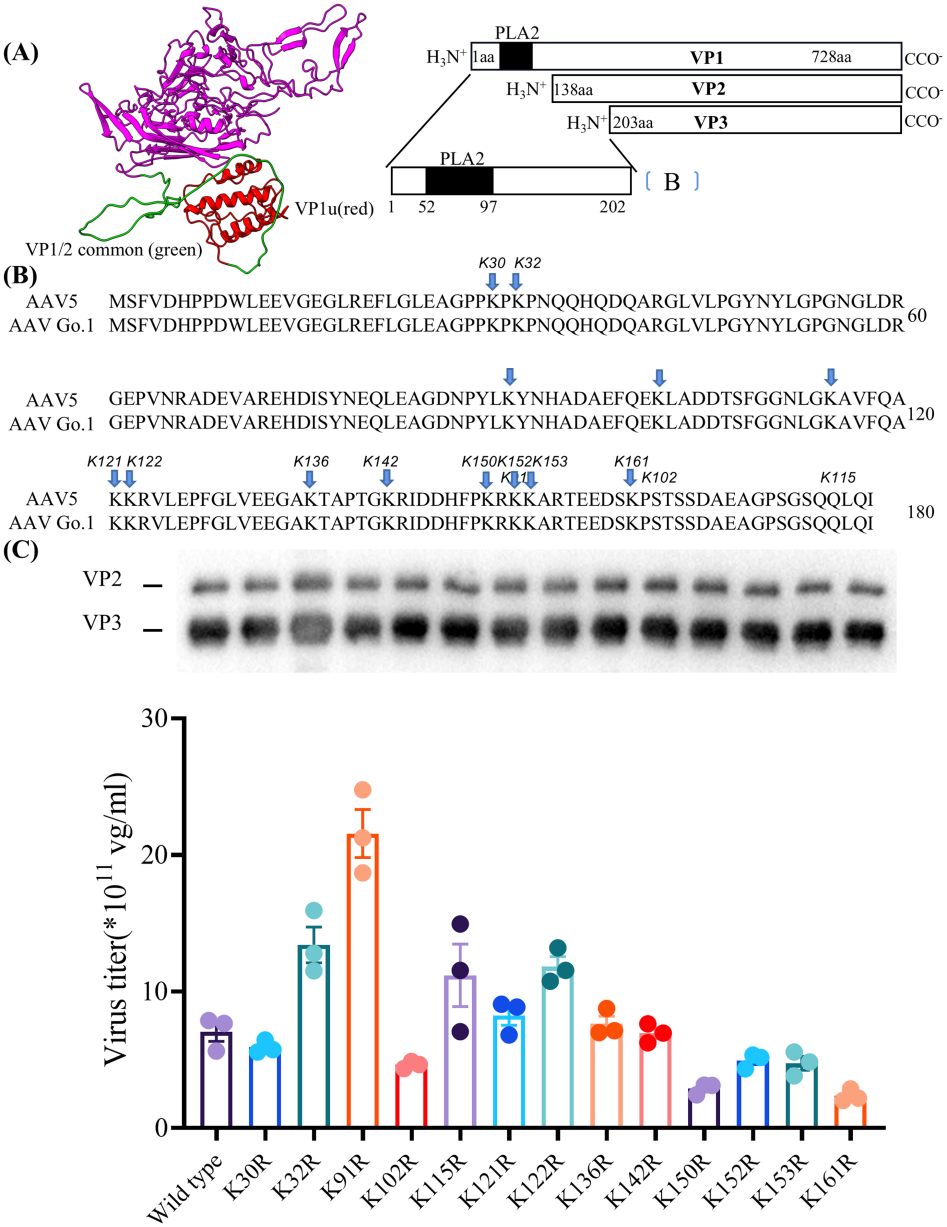
Essential role of lysine residues in the N terminus of VP1 for virus production **(A)** The structure of the VP1 unique VP1u (red) and VP1/2 common region (green) in model. **(B)** Sequence of VP with the positions of the lysine-arginine exchanges highlighted. **(C)** Quantitative PCR results showing the production levels of wild type and different mutant viruses. The dates were shown using mean + SEM for three independent experiments.

For rAAV Go.1, the N termini is conserved among various AAV serotypes and is believed to play a key role in nuclear targeting of the viral genome. Specifically, the N-terminal regions of VP1 and VP2 are critical for AAV infection (Figure 5A). The minor capsid protein VP1 contains a phospholipase A2 (PLA2) domain, which is required for infection at a post-entry step, including endosome release. Proteasome inhibitors can enhance gene transduction. However, as an alternative to inhibitor, point mutations of lysine residues in rAAV VP1 capsid protein have been shown to effectively prevent phosphorylation and subsequent ubiquitination, resulting in significant improved transduction efficiency both *in vitro* and *in vivo*.

Here, the model of rAAV Go.1 sequence is shown in Figure 5A. The VP1 unique region and the VP1/2 common region span amino acids 1 to 203, while the VP3 crystal structure corresponds amino acids 204 to 738. PLA2 is in the VP1 unique structure like other AAVs. We constructed 13 single point mutants in N-terminal VP1 unique and VP1/2 common capsid sequences (Figure 5B) to enhance the potential transduction of rAAV Go.1. To compare the packaging efficiencies between rAAV Go.1 and its 13 single point mutants, vectors containing both GFP and luciferase reporters were packaged using PEI-based triple transfection in 293T cells. The average titers of the 14 vectors from at least three independent packaging experiments were quantified by quantitative PCR after vector purification (Figure 5C). The results indicated that the K91R mutant exhibited a 3-fold higher packaging efficiency compared to the rAAV Go.1 (Figure 5C and Table 2). The mutants K32R and 122R showed 1.9-fold and 1.68-fold improvements, respectively, compared to rAAV Go.1. In the contrast, the K150R and K161R showed lower packaging efficiencies compared to the rAAV Go.1. These findings indicate that the K91R, K32R, and K122R mutants are compatible modifications to the rAAV Go.1 capsid, potentially enhancing its utility for gene therapy applications (Figure 5C and Table 2).

**Table 2:** Effect of the single point mutant on the packing efficiency compared to wild type rAAV Go.1.

Mutant	(Compared to wild type-fold)	
K32R	1.9	(*p* = 0.0131)
K91R	3.06	(*p* = 0.0016)
K122R	1.68	(*p* = 0.0091)
K150R	0.04	(*p* = 0.0052)
K161R	0.33	(*p* = 0.0034)

### 3.6 The rAAV Go.1 mutants-mediated transduction *in vitro* and *in vivo*

**Figure 6:**
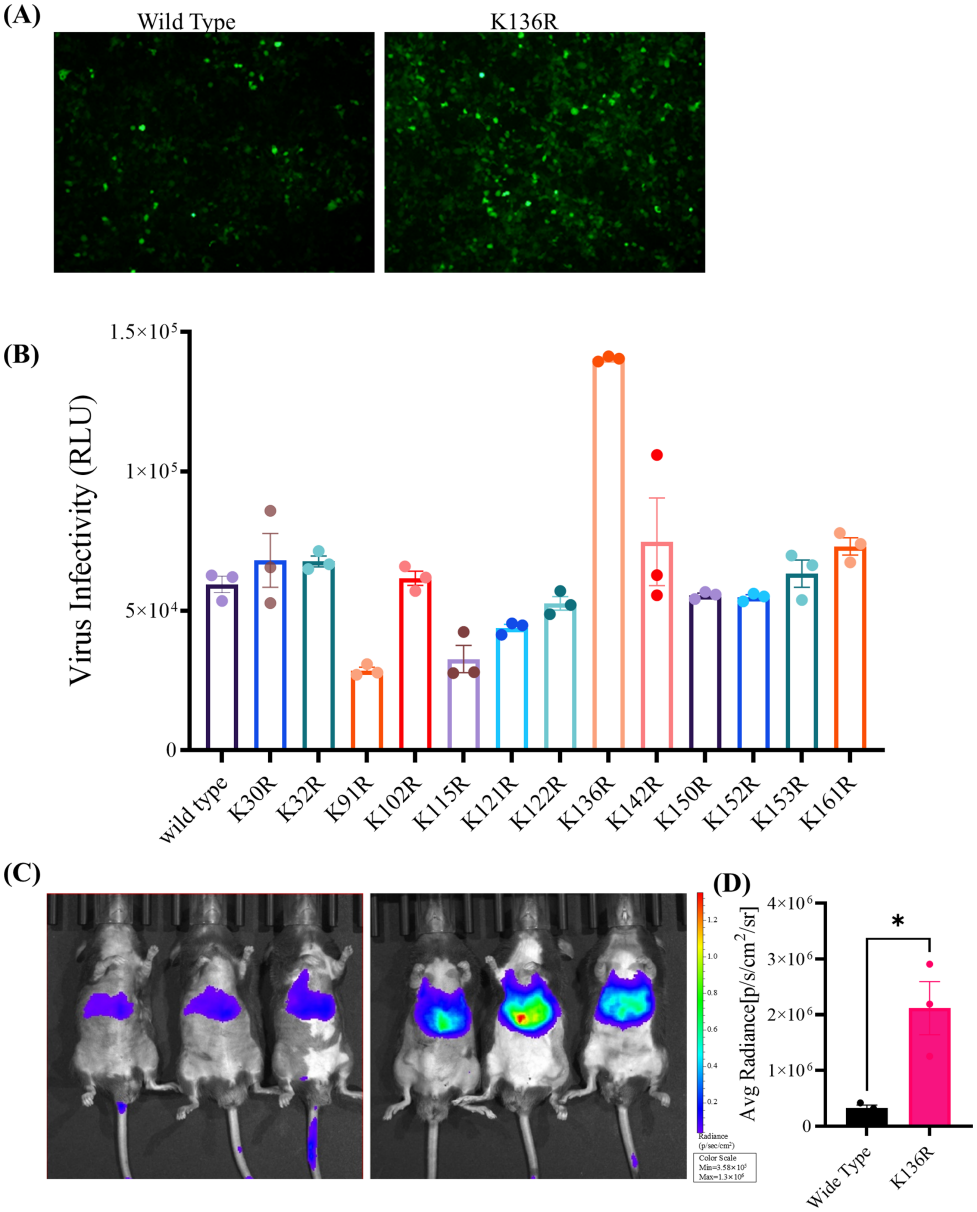
*In vitro and in vivo* transduction efficiency of AAV Go.1 and its mutant vectors. **(A)** HEK293T cells were infected with 10^3^ vg/cell of different AAV Go.1 and various mutant vectors. The green cells were visualized by an Olympus microscrope (Scale bars: 100 μm). **(B)** At 48 h post-infection, cells were lysis and analyzed by luciferase activities. Dates are presented as mean + SEM from three independent experiments. **(C)** *In vivo* imaging of luciferase activity was performed 3 weeks after administration of rAAV Go.1 and its mutant K136R. Representative ventral views of the results are shown. **(D)** Graph showing Average Radiant Efficiency of IVIS imaged mice.

To analyze the transduction potential of these rAAV Go.1 mutants, HEK293T or HeLa cells were infected with 10^5^ vg/cell. At 48 h post-transduction, cells were assessed for luciferase activities. Samples of the immunofluorescence assay were illustrated and compared in Figure 6A. The data showed that the point mutants K136R and K142R could infect more GFP positive cell than the other types of rAAV Go.1 (Figure 6A). The virus infectivity (RLU) was then calculated based on the strengths of the luciferase activities. Results indicated that the point mutants K136R and K142R led to 20–30% enhancement, while K91R, K115R and K121R reduced transduction efficiency compared to the wild type. The remaining point mutations resulted in no significant enhancement of luciferase activities in HEK293T cells (Figure 6B). *In vivo* transduction efficiency was evaluated by injecting 10^11^ vg of each vector into mice via the tail vein. Three weeks post-injection, the mutant K136R achieved significantly higher transgene expression levels compared to wild type (Figures 6C,D). These findings collectively demonstrate that the mutant K136R significantly enhances gene expression in both *in vitro* and *in vivo* models, suggesting its potential as an improved vector for gene therapy.

## 4 Discussions

Here, we characterized AAV Go.1 and analyzed its potential in human gene therapy. A notable feature of this capsid is its high homologous amino acid sequence (94%) with AAV5, which is clearly of human origin and has been isolated from a human penile flat condylomatous lesion (13). AAV Go.1, while highly homologous, is isolated from evolutionarily divergent species. To investigate its suitability for gene therapy, the virus was initially produced using the triple-plasmid transfection method in this study. We found that the previously published plasmid pAAV-RC Go.1, containing AAV Go.1 *rep* and *cap*, had significantly lower virus titer compared to the traditional AAV2 *rep* element and Go.1 *cap* (pAAV-RC2/Go.1). AAV Go.1 with the plasmid pAAV-RC2/Go.1 demonstrated significant improvements in gene expression both *in vitro* and *in vivo*, compared to published rAAV5. The rAAV Go.1 vectors could transduce more cells than rAAV5 vector both *in vitro* and *in vivo*. Biodistribution studies in mice revealed that rAAV Go.1 vectors have a pronounced tropism for kidney and lung, which target the similar organ with rAAV5 (33, 34). Previous studies have reported that rAAV5 and rAAV Go.1 transduce HeLa and 293 T cells with low efficiency, and no significant differences in transduction were observed between the two (19). However, our results demonstrate that rAAV Go.1 exhibits superior transduction efficiency in vitro compared to rAAV5. Moreover, the same viral titer was used to infect both 293 T and HeLa cells. The infection efficiency in HeLa cells, as assessed by the luciferase assay, was 50 times lower, which accounts for the reduced fluorescence intensity observed in the HeLa cell images compared to those of 293 T cells.

The differences in transducing efficiency between AAV Go.1 and AAV5 can be attributed to amino acid differences on their surfaces. AAV Go.1 exhibits stronger binding to human AAVR than AAV5. Specifically, the absence of Ile349 interaction in AAV Go.1, which is present in AAV5 due to Gln532 residue, contributes to this enhanced binding. Furthermore, in AAV5, the Thr712 backbone carbonyl makes contact with AAVR (Arg353), which is absent in AAV Go.1. Furthermore, in AAV5 the Thr_712_ backbone carbonyl makes contact with PKD1, which is absent in AAVGo.1. The largest sequence difference between AAVGo.1 and AAV5 is a tandem serine insertion in VR-V, with AAVGo.1 having a double insertion [L477 (SS)G478], and residues Thr477 to Ser482 in AAVGo.1 are of below-average order in sharpened maps. The variations in amino acid composition indicate that these two viruses were different in receptor usage or other entry functions, with AAV Go.1 enabling more efficient transduction and a lower virus dose, reducing toxicity during treatment (25).

Another challenge in treating human diseases with gene therapy is the presence of AAV neutralizing antibodies, which mediate the rejection of AAV vectors transducing the cells. AAV derived from bat, dog, cat and goat could exhibit poor immunogenicity and low cross-reactivity. When comparing rAAV Go.1 and rAAV5, the 42 amino acid differences are concentrated in the region of VP3 (specifically located from the C terminal of AAV Go.1 to VP1 residue 735), and predominantly on the exterior of the capsid. Some of these 42 amino acids may interact with the footprint of rAAV5 antibodies, and the amino acid variability between rAAV5 and rAAV Go.1 holds functional relevance to capsid-antibody interactions. The low cross-reactivity between anti-rAAV5 antibodies and rAAV Go.1 underscores the latter’s potential as a gene therapy vector in patients with pre-existing immunity to rAAV5. NAb antibody assays demonstrated that AAV5 antibodies exhibit stronger binding to rAAV5 than AAV Go.1. Furthermore, in our *in vitro* assay with health human serum, AAV Go.1 vectors demonstrated greater resistance to neutralization than AAV5 which has already been utilized to treat hemophilia B. The clinical samples were collected from both male and female subjects. Regarding age, we did not specifically stratify the samples based on age, as the primary focus of our study was not to examine age-related differences.

Furthermore, to improve the transduction efficiency of AAV Go.1, the sites of AAV Go.1 (VP1u and VP1/2 common region) were changed from lysine (CTT) to arginine (CCT) in the current study. Since ubiquitination typically occurs on lysine residues, substituting lysine with arginine, a residue with a different side chain, would prevent ubiquitination at that position. This mutation could potentially reduce or eliminate the degradation of the virus by the proteasome, potentially enhancing the virus’s stability and improving transduction efficiency. Results indicated that mutants K91R, K32R and K122R were important for AAV packaging efficiency on the AAV Go.1 capsid. Furthermore, point mutants K136R could led to a 20–30% enhancement in transduction efficiency. However, K91R resulted in reduced transduction, making it unsuitable for gene therapy. The termini of VP1 N and VP2 N in AAV Go.1 contains a phospholipase A2 (PLA) domain and nuclear localization signals (NLS), essential during the infection process at a post-entry step, including endosome release. During the infection process, the VP1 N termini could be exposed to the surface of capsid when pH in the endosome was between 3 and 5 (35). Mutations at these sites could prevent proteasome degradation, allowing more virus to infect cells. Thus, the transducing efficiency of mutant AAV Go.1 was improved.

## 5 Conclusion

In summary, we have constructed AAV capsid from goats, raising questions about the origin and evolution of human rAAV5. The unique tropism and substantial resistance to preexisting humoral immunity exhibited by rAAV Go.1 vectors may render them useful for gene delivery in humans. With their high transducing and packaging efficiencies, rAAV Go.1 could be a better candidate for future gene therapies and gene editing.

## Data Availability

The raw data supporting the conclusions of this article will be made available by the authors, without undue reservation.
